# Minimalism’s Attention Deficit: Distraction, Description, and Mary Robison’s *Why Did I Ever*

**DOI:** 10.1093/alh/ajaa004

**Published:** 2020-05-22

**Authors:** Sophie A Jones

**Affiliations:** Sophie A. Jones is a Wellcome ISSF Postdoctoral Fellow at Birkbeck, University of London, where she works across the fields of post-1945 US literature, gender studies, disability studies, and the critical medical humanities

## Abstract

What does it mean to diagnose a literary work with attention deficit disorder (ADD)? This article traces how US literary minimalism came, in the late twentieth century, to be understood as a literary counterpart to the new diagnostic category of ADD. Pursuing some links between literary criticism and the third volume of the *Diagnostic and Statistical Manual of Mental Disorders*, the article shows how minimalism was seen to resemble the ADD patient because both were defined in terms of a descriptive surface that yielded no depths for expert excavation. Engaging with recent debates on the relative function and value of description and interpretation in literary studies, the article asks whether the notion of an ADD literary aesthetics, grounded in critical disability studies, might provide a route out of the dichotomy of suspicious analysis and reparative description. To pursue this question, the article performs a close reading of Mary Robison’s *Why Did I Ever* (2001), a novel narrated by Money Breton, a woman with an ADD diagnosis. Drawing on the critical disability studies concept of cripistemology, the article shows how Robison’s novel both dismantles the trope of minimalism’s attention deficit and demands a reformulation of the relationship between writing and diagnosis.

In a 1981 essay on his writing process, Raymond Carver attributes his turn to the short story to a crisis of attention:

When I was 27, back in 1966, I found I was having trouble concentrating my attention on long narrative fiction. For a time I experienced difficulty in trying to read it as well as in attempting to write it. My attention span had gone out on me; I no longer had the patience to try to write novels. It's an involved story, too tedious to talk about here. But I know it has much to do now with why I write poems and short stories. Get in, get out. Don't linger.

In Carver’s account, his writing finds its form according to his fluctuating patterns of attention and distraction. Defined in opposition to “long narrative fiction,” his short stories and poems are notable chiefly for their brevity. While Carver’s mode in this essay is primarily introspective, his performance of distracted impatience implicitly raises the question of readerly attention. Is the “involved story” of Carver’s compositional process “too tedious” for him, for his readers, or simply for everyone concerned?^1^

The ambiguous relationship between literary form and that curious dimension of subjectivity known as the attention span was a preoccupation of the US literary world in the 1980s, the period when Carver became the figurehead of a style that came to be constituted—though not without dissent—as US literary minimalism. In a 1985 review, David Leavitt identifies a new group of writers who “have in general limited themselves to the short story, a form they seem to find most appropriate to the age of shortened attention spans, fractured marriages and splintering families in which they grew up.” The following year, John Barth published “A Few Words About Minimalism,” an essay in which he links the mode to “an ever-dwindling readerly attention span,” among other social factors. The cautious generosity of these commentaries about the specific authors under discussion—Barth lists “Frederick Barthelme, Ann Beattie, Raymond Carver, Bobbie Ann Mason, James Robison, Mary Robison and Tobias Wolff” as exemplars of minimalist style—only throws into relief the widespread conviction, consistent throughout these commentaries, that literary minimalism was somehow entangled with the nation’s attention span.

These anxieties over attention spans did not, of course, take place in a hermetically sealed literary culture. In 1980, the third edition of the American Psychiatric Association’s (APA) *Diagnostic and Statistical Manual of Mental Disorders* (*DSM-III*) had introduced a new condition: attention deficit disorder, or ADD. Both broader and more nebulous than the condition it replaced, hyperkinetic reaction of childhood, ADD extended its diagnostic gaze beyond the boisterous boys of popular legend: according to the *DSM-III*, pathological inattention could exist with or without hyperactivity (41). The revised version of the *DSM-III*, published in 1987, drew hyperactivity and inattention into a combined diagnosis, attention deficit hyperactivity disorder (ADHD). Since then, the relative significance of hyperactivity and inattention has continued to be reformulated in subsequent editions of the manual. Under the spotlight of these post-1980 reformulations, a new pool of patients emerged, from the quiet schoolgirl gazing incessantly out of the window to the 40-something underachiever drifting restlessly from job to job.^2^ Notions of pathological attention had been circulating in the medical world for a long time—the Scottish physician Alexander Crichton was writing on “attention, and its diseases” in 1798—but it was from 1980 that the contemporary psychiatric model of disordered attention was codified in the *DSM*, identified in the consulting room, disseminated in the media, and—as I argue here—filtered into literary critical discourse.

How does a neurobehavioral diagnostic category like ADD come to describe literary works as well as patients?^3^ I contend that the trope of “minimalism’s attention deficit” reflects a late twentieth-century insecurity, common to both literature and psychiatry, about objects understood as resisting interpretation. The “minimalism debates” have been well documented. While the mode is closely associated with the editor and teacher Gordon Lish, who (notoriously) steered Carver toward the spare aesthetic for which he is known, the label “minimalist” was in fact rejected by many, if not most of Lish’s authors.^4^ Now, as Margaret Doherty observes, literary minimalism is mainly “noted by literary historians for the heated controversy it generated in newspapers and literary magazines, where defenders and proponents of the movement exchanged salvos throughout the 1980s” (88). Less well-documented are the ways in which these debates positioned minimalist writing as an affront to the project of literary hermeneutics itself. According to its detractors, literary minimalism resisted interpretation not to advance the interest of avant-garde difficulty (which might have been acceptable) but to disguise its meaninglessness and triviality—qualities that rendered the task of literary interpretation precarious, perhaps even moot. Here I neither intend to enter into the minimalism debates nor seek to define or defend the term’s utility as a category of literary analysis. Instead, I approach US literary minimalism as an object constituted through the various screeds, dismissals, defenses, and curious probings of authors, journalists, and academic critics in the late twentieth century.

The essays, articles, and reviews that helped to establish literary minimalism draw frequently on notions of attention deficit, a concept that was itself bound up with a debate about the fate of interpretation and analysis at the end of the twentieth century. ADD was codified as a condition just as the psychiatric profession shifted its allegiance from psychoanalysis to descriptive psychiatry. Within the terms of this new paradigm, the attention deficit patient would be diagnosed and treated according to a standardized list of observable symptoms, rather than analyzed by an individual expert. The trope of minimalism’s attention deficit, in this context, was never a simple slur: literary minimalism was seen to resemble the ADD patient, not simply because both were in deficit, but because both were defined in terms of a descriptive surface that yielded no hidden depths for expert excavation.

[L]iterary minimalism was seen to resemble the ADD patient . . . because both were defined in terms of a descriptive surface that yielded no hidden depths for expert excavation.

Such concerns resonate with recent debates about the relative function and value of interpretation and description in literary studies. While minimalism’s critics saw the mode’s descriptive tendencies as a sign of its deficient attention span, recent work by Heather Love, Sharon Marcus, and Stephen Best has emphasized the power of literary description to repair or enhance attention. A revaluation of the terms of the minimalism debates is evident, albeit implicitly, in this so-called descriptive turn in literary studies. Witness, for instance, Love’s endorsement of a “minimalist but painstaking work of description” that “undermines the ethical charisma of the critic” (387). Here we can see anxieties around the waning of interpretive authority refigured as strengths: Love suggests that critics should *become* minimalists by embracing description. Using similar language in their article “Surface Reading,” Best and Marcus identify description with a practice of “minimal critical agency” and a tendency to be “relatively neutral” about the object of study (17, 16).

This scholarly work on description is one dimension of a broader movement variously termed the post-critical or post-hermeneutic turn. For Rita Felski and Elizabeth S. Anker, this turn involves a self-conscious shift away from a “*diagnostic* quality of critique” grounded in psychoanalysis (Anker and Felski 4). Just as Sigmund Freud analyzed his patients, so too “a generation of critics scrutinized literary and cultural texts for their accidental or involuntary betrayal of repressed meanings,” argues Felski (61–62). What Felski, after Paul Ricoeur, calls the “hermeneutics of suspicion” has, perhaps, informed existing scholarly analyses of minimalism as a literary negotiation with trauma (9).^5^ I here argue that, in the context of literary minimalism, trauma and ADD have functioned as alternative paradigms for the act Felski terms “diagnostic” reading. Where the trauma paradigm finds hidden etiological depths in minimalist literature, the ADD paradigm sees only the symptomatic surface. In this context, description is aligned with diagnostic reading too. One question that I derive from these debates is whether the notion of an ADD literary aesthetics, grounded in a critical disability studies that is far from neutral, might provide a route out of the dichotomy of analysis and description—and thus model a new way of understanding the relationship between literature and psychiatric diagnosis.

To pursue this question, I turn in the latter part of this article from criticism of minimalism to the literature itself. In particular, I read Mary Robison, a paradigmatic (if reluctant) minimalist whose 2001 work *Why Did I Ever: A Novel* dismantles the minimalism’s attention deficit trope. My reading of Robison’s novel draws on the notion of cripistemology, as put forward by disability theorists Merri Lisa Johnson, Robert McRuer, and Lisa Duggan, to propose a new way of conceptualizing the relationships connecting attention disorders, trauma, and minimalist literature. Moving away from the opposition between analytic depth and surface description, I suggest cripistemological reading as a framework for thinking through Robison’s literary negotiation with neurobehavioral disability. To begin, however, it is necessary to return to the origins of the charge of minimalism’s attention deficit: in literary debates of the 1980s and 1990s.

## 1. Forms of Inscrutability: Literary Minimalism and ADD

Depthless, placeless, hyperreal: the adjectives often attached to minimalist writing are common to broader assessments of postmodernism. Contemporary commentators often drew parallels, implicit or explicit, between literary minimalism’s affective flatness and postmodern culture’s move away from depth models, as Fredric Jameson would have it. Barth, the canonical metafictionalist, draws minimalism into the orbit of postmodernism when he states that the mode has been both “praised and damned under such labels as ‘K-Mart realism,’ ‘hick chic,’ ‘Diet-Pepsi minimalism’ and ‘post-Vietnam, post-literary, postmodernist blue-collar neo-early-Hemingwayism.’” Thus, while recent scholarship has often positioned minimalism as a realist counterpoint to the kind of postmodern metafiction produced by Barth and his peers, its contemporaneous reception points to a more complex negotiation with the postmodern.^6^ If the author of postmodern metafiction is displaced by the text, then the author of minimalist fiction is displaced differently: according to their critics, minimalist authors are not dead but in a state of cognitive deficit.

This idea is at work in fiction writer John Biguenet’s fragmented 1985 essay “Notes of a Disaffected Reader,” which adopts the telegraphic style it critiques. Biguenet asserts, “The abrupt sentence forms the basic unit of minimalist prose. It is syntax without an attention span” (42). His phrasing makes the text, rather than the writer, the bearer of deficiency—an appropriate rhetorical move, given that his broader argument associates minimalist literature with poststructuralist theories that decenter the author. Biguenet declares that minimalists “are the slaves of Derrida”: in relinquishing their status as authors, he contends, they retreat into solipsistic paraphrase (45). As a result, “[t]he reader, like a child with crayons hunched over a coloring book, authors the story” (44). Significantly, the reader who displaces the author is here configured as an intellectually inferior child, perhaps even the kind who, by the mid-1980s, might well have been diagnosed with ADD.

Barth is more ambivalent and sometimes even hopeful about literary minimalism’s stylistic value, but the association of minimalist literature with cognitive deficit still rings loud and clear in his article. Noting a “national decline in reading and writing skills” that has percolated into graduate writing programs, Barth elaborates:

Rarely in their own writing, whatever its considerable other merits, will one find a sentence of any syntactical complexity, for example, and inasmuch as a language’s repertoire of other-than-basic syntactical devices permits its users to articulate other-than-basic thoughts and feelings, Dick-and-Jane prose tends to be emotionally and intellectually poorer than Henry James prose. Among the great minimalist writers, this impoverishment is elected and strategic: simplification in the interest of strength, or of some other value. Among the less great it may be faute de mieux. Among today’s “common readers” it is pandemic. Along with this decline, an ever-dwindling readerly attention span.

Barth’s ambivalent assessment of minimalism tips over into critique thanks to his style, which performs the “syntactical complexity” against which the minimalists are assessed and (often) found lacking. Meanwhile, his reference to “Dick-and-Jane prose” recalls Biguenet’s imbrication of minimalism and infancy. For Barth, the understated prose of minimalist literature presents a problem for the critic: how, in the absence of “Henry James prose,” is one to tell the difference between simplicity and childishness, or style and deficit? The directness of minimalist style, he suggests, makes it ever more difficult to distinguish great writers from mediocre imitators—a problem exacerbated by the impoverished attention span of the (childish) common reader.

If Biguenet and Barth were concerned with the abdication of authorial primacy in minimalist writing, some commentators were vexed by what they saw as the diminishment of critical authority augured by minimalism. In his *Talents and Technicians: Literary Chic and the New Assembly-Line Fiction* (1992), John W. Aldridge, for one, bemoans the waning influence of literary criticism. The problem, for Aldridge, can be traced to a literary market that obstructs “the symbiotic relationship between serious criticism and the production of literature” and instead treats writing like an assembly line (8). But, Aldridge says, the fault also lies with minimalist literature: a writer like Amy Hempel, he charges, “offers us too little evidence for interpretation” (72). Taking aim at minimalism’s frayed link between evidence and interpretation, Aldridge suggests that its authors obstruct the critic’s main objective: to analyze.

In the 1980s, psychiatry was entangled in its own set of debates about hermeneutic models of knowledge. The *DSM-III*, which introduced ADD as a diagnosis, represented a major sea change in psychiatric practice as the manual moved away from psychoanalysis and adopted a descriptive approach chiefly concerned with identifying symptoms, not interpreting them to discover their causes. The volume explained in its introduction that, because most of the disorders it listed had unknown causes, it would adopt an approach that was largely “atheoretical with regard to etiology or pathophysiological process” (*DSM-III* 7). Where the *DSM-II* (1968) had explicitly signaled causation with its term *hyperkinetic* reaction *of childhood* (50; emphasis added), the *DSM-III* offered a new “atheoretical” term for the condition: *attention deficit disorder*. The volume’s longer list of diagnostic criteria for the newly conceptualized condition restricted its focus to behavioral symptoms, such as “often fails to finish things he or she starts” and “frequently calls out in class” (43–44).

The *DSM*’s turn to descriptive psychiatry was controversial. It gave psychiatrists “an agreed-upon language for naming what they saw, yet this language did not explicitly engage the issue of the nature of the disorders it named,” according to the psychoanalytic psychiatrist Mitchell Wilson, who observes that “while the clarity of the language facilitated communication, it did so by bracketing what had hitherto been essential clinical concepts in psychiatry.” One effect of this bracketing is, he adds, a “narrowing of the psychiatric gaze” marked by a “loss of the concept of depth of mind, a loss of the concept of the unconscious” (408).

This charge of a “narrow[ed]” psychiatric gaze resonates with the critiques leveled at literary minimalism during the same period. Just as psychoanalytic psychiatrists saw the *DSM-III* as an attack on their authoritative clinical judgment, so did some literary critics and authors see minimalist literature as an affront to their autonomy. The shift to descriptive psychiatry appeared to challenge the authority of the individual clinician, whose professional opinion counted for much less in an era of standardized classification. According to the psychiatrist Howard Berk, “the *DSM-III* gets rid of the castle of neurosis and replaces it with a diagnostic Levittown” (qtd. in Spiegel). Berk’s parallel between the standardized housing of the postwar suburban development and the standardized diagnostic categories of the *DSM-III* recalls Aldridge’s notion of “assembly-line fiction”: both literary critic and psychiatrist claim to bear witness to a new era in which a systematizing approach is seen as replacing professional discernment and judgment. This new approach to psychiatric assessment entailed a new account of the symptom. As Jennifer Fleissner has noted, there is a radical difference between Freud’s suggestion that “it is difficult to attribute too much sense to [the symptom]” and neuropsychiatry’s tendency to “strongly reject the notion of attributing any deep hermeneutic significance to the individual symptom” (387, 388). Fleissner articulates a question that remains coded in the dominant critiques of minimalism: What happens to literature when the symptom can no longer be interpreted? Literary minimalism was constituted as an object of critical appraisal in the shadow of this question.

The insecure status of the critic in an age of meaningless symptoms is an evident concern in the novelist Madison Smartt Bell’s scathing 1986 *Harper’s Magazine* essay on minimalism, “Less is Less: The Dwindling American Short Story,” an influential article on which Aldridge, among others, draws. Bemoaning minimalist literature’s “obsessive concern for surface detail,” Bell describes Leavitt’s comment connecting the short story to waning attention spans as “perturbing” (65). In contrasting the new short story with its forebears, Bell complains that, while Ernest Hemingway constructed entire life histories for his characters, which then became resounding absences in his prose, in the minimalist short story, “the unspoken has simply been left unthought” (66). This comment might just as well critique descriptive psychiatry’s concern with surface symptoms and lack of interest in their unconscious roots. Yet, interestingly, Bell illustrates his castigation of minimalism with an exception that garners his rare, if muted, praise: Hempel’s “In the Cemetery Where Al Jolson Is Buried,” from her debut collection *Reasons to Live* (1985).

In Hempel’s story, the narrator recounts the time she spent with her dying best friend in a hospital. The narrator, at her friend’s behest, distracts her with trivia:

“Tell me things I won’t mind forgetting,” she said. “Make it useless stuff or skip it.”

I began. I told her insects fly through rain, missing every drop, never getting wet. I told her no one in America owned a tape recorder before Bing Crosby did. I told her the shape of the moon is like a banana—you see it looking full, you’re seeing it end-on. (29)

Bell exempts this story from his broader critique of minimalism because its “strategy of distraction” eventually “rises above the trivial” to symbolize the narrator’s inability to confront her friend’s death (66). In Hempel’s other stories, he maintains, “the trivial remains just that”: a meaningless aesthetic of distraction that resists the act of interpretation (66). For Bell, then, the trivial is acceptable only when it conceals a trauma that the critic can probe. The idea that language might be reduced to a meaningless symptom, closed to interpretation, is what troubles Bell about minimalism. By making an exception of Hempel’s story, Bell identifies the other works under discussion, including the remaining stories in *Reasons to Live*, with a crisis of interpretability engendered by a body of writing that, like the symptoms of attention disorders, can be read but not analyzed.

Trauma is one of the multiple factors Barth lists as possible contributors to the emergence of minimalism. The “national hangover from the Vietnam War,” he explains, was “felt by many to be a trauma literally and figuratively unspeakable” and thus amenable to the opacity of minimalist prose. In subsequent decades, the traumatic reading of minimalism came to dominate scholarly criticism of the mode. Indeed, theories of trauma loomed large in the broader field of literary scholarship from the 1990s onward, following the *DSM-III*’s introduction of posttraumatic stress disorder (PTSD) as a discrete category. This development, notes Ruth Leys in *Trauma: A Genealogy* (2000), was part of “an essentially political struggle by psychiatrists, social workers, activists and others to acknowledge the postwar sufferings of the Vietnam War veteran,” as well as the long-term effects of childhood sexual abuse (5). During this period, a poststructuralist academic model of trauma was forged that resisted dichotomizing the psychoanalytic and the neuropsychiatric paradigms. This model, most powerfully associated with the work of Cathy Caruth, emphasizes the paradox of trauma: “that the most direct seeing of a violent event may occur as an absolute inability to know it; that immediacy, paradoxically, may take the form of belatedness” (94).

This statement points to what notions of traumatized minimalism have in common with notions of minimalism’s attention deficit. Both tropes use psychiatric terminology to make sense of the relationship between surface and depth, or the known and the unknown, in literature. Yet trauma, an established paradigm in academic literary criticism, does not carry the derogatory charge of ADD. There are important differences between the two diagnoses: while the capacious category of trauma has allowed Caruth to integrate the psychiatric model of PTSD with earlier psychoanalytic frameworks, attention disorders are more narrowly aligned with the post-1980 paradigm of descriptive psychiatry. If trauma preserves an interpretive role for both the literary critic and the psychoanalyst, ADD presents an opaque face marked with what Fleissner might term senseless symptoms. Thus, for Bell, Hempel’s only good story is the one in which distraction is transformed into an expression of trauma—a trauma that gives the reader, as well as the psychotherapist, something to do.

The tension between these paradigms in the wider culture is on display in a landmark narrative of adult ADD diagnosis, Frank Wolkenberg’s 1987 *New York Times* article “Out of a Darkness.” Here Wolkenberg describes his “overwhelming relief” once he had been diagnosed with ADD. He had previously suspected that his problems were “linked to [his] mother's death when [he] was 12, and to hearing, as a child, about [his] family’s experiences during the Holocaust.” He also expresses relief at the news that his difficulties with organization, focus, and mood swings do not signify unconscious traumatic experience; they refer, instead, to what he calls “a dysfunction of genetic origin, most likely a chemical failure in the system of the brain.” Wolkenberg comes to know himself as a “neurochemical self,” to borrow Nikolas Rose’s term, and he is reassured by this new understanding of his behavior because, unlike the trauma of a mother’s death, neurochemistry can be altered with the help of Ritalin (Rose 46). Wolkenberg acknowledges that the symptoms of ADD are usually associated with childhood but takes note that “specialists now believe that it affects at least 2 million American adults,” despite the lack of “conclusive data” about the condition.

It is significant that Wolkenberg’s desired brain scans that would reveal the mysteries of attention disorders were not yet a reality in 1987—indeed, by the twenty-first century, ADHD diagnoses still depend on behavioral assessments rather than neuroimaging. That the “chemical failure” thought to underlie attention disorders could not be detected by tests or scans was, in a way, beside the point in light of the *DSM-III*’s descriptive approach, which left a blank space for scientific speculation about the “true cause” of ADD. The introduction to the *DSM-III* propounds the radical democracy of descriptive psychiatry. It assures readers that “[t]he major justification for the generally atheoretical approach taken in *DSM-III* with regard to etiology is that the inclusion of etiological theories would be an obstacle to use of the manual by clinicians of varying theoretical orientations, since it would not be possible to present all reasonable etiological theories for each disorder” (7). Yet the volume was published during the ascent to dominance of biological psychiatry; its expulsion of psychoanalytic terminology was seen by some to give free rein to biological approaches, as Matthew Smith has observed (97). Meanwhile, a close alliance had developed between attention disorder research and pharmaceutical firms: Ciba, the manufacturer of Ritalin, directed money to ADD research and patient advocacy organizations, as well as advertising directly to psychiatric practitioners (Smith 94–95; Schwarz 108–9). Despite its claims to neutrality, the *DSM*’s descriptive psychiatry seemed particularly well suited to act as scaffolding for neurobiological approaches.

Bound to the promissory structure of neurochemistry, accounts of attention disorders in the post-1980 period turn the paradox of trauma inside out: they gesture toward inaccessible knowledge that beckons from the future, not from the past. The knowledge that would account for trauma lies in the past, even if that past cannot be comprehended; conversely, the knowledge that would explain attention disorders is always pending. This point is crucial: within the parameters forged by the *DSM*, it is not that attention disorders *cannot* be fully known, but that they are *not yet* fully known. As the psychiatrist Jerrold Maxmen puts it in his *The New Psychiatry* (1985), “*DSM-III* spotlights the enormous gaps in factual information about mental disorders. From now on, a major goal of psychiatric research is to fill these gaps” (58). As Maxmen’s comments demonstrate, the *DSM*’s concerted marking of its own epistemological limits points toward their future transcendence. The minimalism of descriptive psychiatry produces a blank space for scientific speculation; this is the space to which Wolkenberg appeals when he speaks of the brain scans that will one day reveal the mysteries of ADD. This context indicates how trauma and ADD have functioned as divergent conceptual frameworks for making sense of literary minimalism’s opacity. Minimalism became the stage for an uneasy collision of psychiatric paradigms focused on inaccessible knowledge but with different temporal inclinations. If trauma theory posits a crisis of representation whereby the past becomes inaccessible to memory and expressible only in terms of the mute symptom, ADD’s mute symptoms have no history at all—only a future. 

At this point, it is worth briefly returning to Hempel and the conception of knowledge established in “In the Cemetery Where Al Jolson is Buried.” Bell’s preoccupation with the traumatic origin of the trivial information in Hempel’s story sidesteps the important fact that much of this information is erroneous. The dying friend, because she is dying, does not care whether the information she receives is true or false. Indeed, she specifically requests “useless stuff” that she “won’t mind forgetting.” For Hempel’s narrator, the unseen part of the moon prompts a whimsical story, not a quest for the truth. There is an important difference between the strategically erroneous “information” in Hempel’s story, which positions itself against the quest for verisimilitude, and the “description” of the *DSM*, which takes great care to delineate the known, which can be stated, from the unknown, which cannot. 

I would suggest that the pursuit of certainty implicit in the *DSM-III*’s descriptive psychiatry can be compared to recent defenses of the value of description in literary studies. Working in the orbit of the postcritical turn, Marcus, Love, and Best, among others, have addressed what they see as the unjust denigration of both creative and critical literary description. In their coauthored article “Building a Better Description,” they suggest incorporating “the uncertainty of any attempt to describe into descriptions themselves” (10). The descriptive psychiatry of the *DSM* arguably does just that: the volume’s exclusion of etiology embeds gaps in psychiatric knowledge into its diagnostic descriptions. This acknowledgment of uncertainty, however, coexists with a commitment to the pursuit of certainty within a neurobiological paradigm. The idealized neutrality of description, supposedly embedded in its necessary incompletion, gives way in this case to an investment in imagined future certainty. The act of circumscribing the limits of knowledge about a psychiatric condition like ADD works, counterintuitively, to sustain a fantasy of future neurobiological certainty and psychopharmaceutical correction.

A further parallel might be drawn between the descriptive psychiatry of the *DSM* and the descriptive turn in literary studies: a commitment to overcoming deficits of attention. Marcus, Love, and Best mention the reparative powers of attention several times in “Building a Better Description.” In response to the question, “Why describe?” they assert:

Because describing and descriptions can produce pleasure—granular, slow, compressed, *attentive*, appreciative—as when Roland Barthes reproduces, codes, and interprets every sentence of a Balzac novella in *S/Z*, then reproduces the text again in its entirety.* Because description can make us more attentive*, as when we produce an audio description, copy a painting, analyze or perform a piece of music, and annotate or memorize a text. *Because description can allow us both to see more and to look more attentively*, more fully, and more selectively. (14; emphasis added)

This is a departure from earlier endorsements of critical description, such as Susan Sontag’s in her 1964 essay “Against Interpretation.” If Sontag is primarily concerned with doing justice to aesthetic form, Marcus, Love, and Best are interested in the ethical disposition of the critic. Description, in this account, is not diagnostic but therapeutic. Prized as an ethical attribute, description becomes a form of cognitive enhancement or psychological therapy, automatically engendering better habits of attention. This account of description resonates with the *DSM*’s approach: while the manual is not itself therapeutic, the APA implicitly identifies descriptive minimalism with a neutrality that (in its view) can engender successful treatment.

If the turn to literary description replaces diagnostic reading with therapeutic reading, what happens to ADD? Is it possible to speak of an ADD aesthetics that avoids both the derogatory charge of diagnostic reading *and* the therapeutic promise of descriptive reading? One approach to such a challenge can be found in the work of Robison, who has (to her professed dismay) been marketed and read as a minimalist since the late 1970s. Robison’s 2001 novel *Why Did I Ever* involves a parodic, critical return to the minimalism’s attention deficit trope. The novel, I contend, engages with the relationship between trauma and ADD without forcing these conditions into a binary framework that opposes the unchangeable traumatic past to the correctable neurobiological future. Instead, Robison adopts a form of writing that I call cripistemological, adopting a term coined by Lisa Duggan and further theorized by Johnson, McRuer, Jasbir Puar, and others.

“Cripistemology,” or “thinking from the critical, social, and personal position of disability” (Johnson and McRuer, “Cripistemologies” 134), involves, in Puar’s words:

a critique of the notion of epistemology itself, a displacement not only of conventional ways of knowing and organizing knowledge, but also of the mandate of knowing itself, of the consolidation of knowledge. This supplements a cripistemology with “crip(s) at the beginning or center of the production of knowledge” by offering another reading of cripistemology as a matter of debilitating contemporary forms of knowing with forms of unknowing, sensing, refusing to know, akin to Jack’s formulation, and, further, a matter of challenging the status of knowledge itself. (qtd. in Johnson and McRuer, “Proliferating Cripistemologies” 163–64)^7^

Puar’s intervention here points to the value of cripistemology, and critical disability studies more widely, as an alternative to the interpretation/description binary. Minimalist literature was framed as a mode of attention deficit writing because it was seen to resist forms of interpretive knowledge. The clinical account of attention disorders, however, cannot be said to resist “the mandate of knowing itself”; rather, the *DSM* points out the gaps in knowledge in order that psychiatric research might fill them. Robison’s *Why Did I Ever*, in contrast, performs Puar’s project of “debilitating contemporary forms of knowing with forms of unknowing.” In so doing, Robison refigures the interaction of trauma and ADD through a cripistemological lens.

## 2. Mary Robison’s Post-Minimalist Cripistemology

Robison came to prominence as a writer of short fiction in the late 1970s and, to date, she has published four collections of stories and four novels. *Why Did I Ever*, her third novel, is narrated by Money Breton, a woman with ADD. Before I turn to it, I want to consider Robison’s initial hailing as a founding minimalist author in the late 1970s and 1980s. Anatole Broyard’s review of her first short story collection, *Days* (1979), exemplifies Robison’s reception. Deploying a familiar vocabulary of inscrutability, uninterpretability, and minimal scale, Broyard wonders “whether it is a virtue of Miss Robison to have offered me a novel experience or whether she has simply imposed on me a scale of values that I cannot interpret.” He continues, “Some of Miss Robison’s sentences achieve a rather impressive inscrutability.” Finally, he admits: “I’m divided in my mind whether to be grateful to Miss Robison for calling my attention to the minuscule mysteries that make up the nitty-gritty of our anxieties, or whether to see her as one of those people who root around in trash cans.” Setting the tone for a whole generation of critical evaluations of minimalism, Broyard all but wonders: Is the literature deficient or am I?

Robison herself rejects the term *minimalism*. As she puts it in a 2001 interview:

I detested it. Subtractionist, I preferred. That at least implied a little effort. Minimalists sounded like we had tiny vocabularies and few ways to use the few words we knew. I thought the term was demeaning; reductive, clouded, misleading, lazily borrowed from painting and that it should have been put back where it belonged. (M. Murray)

It is notable that Robison discusses minimalism in the past tense: indeed, by 2001, the moment of US literary minimalism was widely seen to have passed. *Why Did I Ever*, Robison’s first publication since 1991’s *Subtraction*, marked the end of a decade of writer’s block; with it, she launched her latest work into a new literary era, marked, in part, by the success of maximalist American novels by the likes of David Foster Wallace and Jonathan Franzen.^8^

Yet *Why Did I Ever* was a milestone for another reason. Robison had moved from Knopf, which published her first five books under Gordon Lish’s editorship, to Counterpoint Press, with which she published two more books following *Why Did I Ever*: the short story collection *Tell Me* (2002) and *One D.O.A, One on the Way* (2009), a novel set in post-Katrina New Orleans. *Tell Me* bears traces of Robison’s fraught relationship with Lish: the collection is purged of his editorial contributions, restoring the original stories as they were edited by Roger Angell for *The New Yorker*.^9^ “The differences were significant to me,” Robison stated in a 2003 interview with Michael Silverblatt on his radio show *Bookworm* (1989–), where she expressed regret at the number of anthologies featuring the Lish-edited versions of these early works.

Yet Robison’s post-2000 output is not only a project of restoration. Her two recent novels write back to the moment of minimalism, subverting the aesthetic of radical cutting associated with Lish’s editorial practice and reworking the metaphors of attention and distraction through which minimalism was constituted. In a recent appreciation of *Why Did I Ever*, Blake Butler proclaims that the novel distinguishes itself from “so much modern writing these days trying to find a way to explain our situation as plush but dire, free but under surveillance, exhausted but ADD.” Using ADD adjectivally, Butler echoes the doomsaying about contemporary attention spans that characterized the 1980s emergence of minimalism. Yet *Why Did I Ever’*s interest in ADD runs far deeper than Butler’s comment recognizes. Robison, who has discussed her own ADD diagnosis in interviews, gives the condition to her protagonist, Money, as well. Literalizing the critical trope of minimalism’s attention deficit, Robison practices a cripistemological revision of its premises.

While Robison’s screwball dialogue and oblique narration are consistent across her body of work, *Why Did I Ever* represents a distinct stylistic shift. Her early fiction, which has been described as hyperreal, displays a formal fidelity to the traditions of the novel and short story.^10^*Why Did I Ever*, in contrast, is structurally fragmented. Subtitled *A Novel* (all Robison’s books announce their genre in their subtitles), it is, arguably, not immediately recognizable as one. The book is divided into 536 short sections—some numbered; others titled with what seem like excerpts of ambient conversation drawn from Money’s chaotic life, such as “*And Yet*,” “*And Then a Kitchen Fire*,” and “*This Was Your Idea.*”

Composed on a series of index cards after a period of writer’s block, *Why Did I Ever* was probably not what Barth was thinking of when he reminisced about the sustained attention that novels demand.^11^ Indeed, Robison has publicly linked the book’s episodic structure to the willed state of distraction in which it was composed. In the same interview quoted above, she contextualizes the book’s composition:

[V]arious horrible things had happened, as they sometimes will, and I was having difficulty. I was having more than difficulty. Like a repulsive videotape was on automatic replay in my head. So to get through, I began scribbling notes. I would go out, take a notebook. Or drive, or park wherever and take notes. I would note anything left. Anything that still seemed funny or scary or involving for four seconds. Some berserk conversation I overheard. The crap on the radio. This big, brilliant cat. Ridiculous weather. Then it was months before I read over the scribbles and realized they had a steady voice, and that there were characters and themes. Although none of the material was organized at all except around my urgent need to distract myself. (M. Murray)

Distraction, Robison tells us, is more than just the subject of the novel, operating also as a structuring principle during its composition. Described by the author as a process of assembly, the novel’s composition involved the numbering and arranging of these fragmented “scribbles” into a narrative. While Robison’s reference to assembly recalls Aldridge’s caricature of standardized “assembly-line fiction,” her idiosyncratic process generates a more complex vision of what it might mean—or fail to mean—to write in a state of distraction.

Robison’s description of feeling as if “a repulsive videotape was on automatic replay in [her] head” blurs the boundary between distracted and traumatic experience. Echoing her account of its process of composition, *Why Did I Ever* explores the interrelation of trauma and ADD in the life of its narrator, Money, whose son, Paulie, is recovering from a violent sexual assault and whose daughter, Mev, is addicted to methadone. The novel, by exploring Money’s distress as she confronts what has happened to Paulie, represents the complex entanglement of attention disorders and traumatic experience without seeking a final explanation for their interrelation. This refusal to dichotomize trauma and ADD emerges particularly in passages dealing with Money’s troubled relationship with the ADD drug Ritalin. On a break during one of her long, insomniac night drives, Money mulls her predicament:

I take the corner booth at IHOP, where perhaps I can last until two. Thinking about my lean and suntanned son. Weeping into a napkin. Ignoring a short stack and a side of links that, anyway, would be tastier if I ate their depiction on the menu.

I have long thought pharmaceutical drugs were the solution and I was right about that and that’s correct. Still, you have to consider, with even the best prescription drugs, who it is who’s taking them. (97)

Here the novel is not concerned with debunking pharmaceutical treatments or suggesting that ADD is an industrially conjured myth: in a conversation with her mother, Money describes the condition as a “birth defect” that she hasn’t “been able to shake” (37). Rather, it uses Money’s growing ambivalence about her use of Ritalin to interrogate the conditions under which pharmaceutical interventions might fail to address the specific circumstances—and, specifically, the traumatic experience—of “who it is who’s taking them.”

*Why Did I Ever* explores what happens when, as a result of trauma, there is no desire or capacity for heightened focus. Instead, there is an “urgent need to distract” oneself, to borrow Robison’s words about her experience of writing the novel. The novel pursues this question by showing how Money’s distress transforms the way she inhabits her ADD diagnosis, opening up a space between her experience and dominant clinical accounts of what it means to be impaired by an attention disorder. Such accounts tend to emphasize economic productivity. Since the *DSM-III*, the APA has explicitly and repeatedly contextualized attention deficit behavior with reference to its implications for work, whether at school or in employment. *Why Did I Ever* both echoes and subverts the *DSM*’s emphasis on productivity. A precariously employed script editor, Money acknowledges that her quirks have lost her contracts with studios, admitting that “they had to *sit* on me to get me to work” (88). She also observes, however, that her colleagues both stigmatize and fetishize her condition:

The only thing I really have going for me is my attention deficit. It’s very, very impressive to these people. How I forget to collect my checks, or fail to kiss the ring of whichever the hell one is the studio president.

On the debit side, I missed removing an electric roller this morning and did the sushi lunch and the studio meeting with it lodged in the back of my hair. (121)

Money’s caustic accounting of her condition’s credits and debits reflects the pressure neoliberal capitalism exerts on ways of conceptualizing attention disorders, a pressure that increasingly searches for the condition’s economic advantages as well as its deficits. As Stuart Murray has observed, “a culture of work acceleration and multiple-project multitasking or, conversely, sustained concentration and single tasking, might seem to welcome the forms of cognitive *variation* inherent in some neurobehavioral conditions, such as autism.” In the case of attention disorders, popular psychologists such as Edward Hallowell have drawn attention to the advantages wrought by the states of “hyperfocus” experienced by some people with the condition (177–78).

The limits of psychiatric knowledge of attention disorders create a space for speculation not only about the profession’s future knowledge of the condition but also about the individual patient’s economically productive posttreatment future. In the contemporary moment, the “deficit” of ADHD has been reframed as an opportunity not only for treatment but also for enhancement, and the stimulants used to treat the condition are lauded for their ability to augment productivity rates, even for those who have not received a diagnosis. In *Why Did I Ever*, Money opts out of such dreams of more and better labor, while yearning to be free of work altogether: “If I could take a break from work I could read all my books, contact everyone, clean everything, learn to play the drums, drive to Quebec, Canada, and I would try to come back right away” (182). The passage tests what it might mean to conceptualize attention disorders without reference to their impact on economic productivity.

With its representation of the imbrication of Money’s ADD with her working life, *Why Did I Ever* engages with the problem of what it means to diagnose ADD when the horizons of psychiatric knowledge are necessarily conditioned by capitalist economic imperatives. Johnson and McRuer introduce the concept of cripistemology by discussing the forms of knowledge that neoliberal health economies produce: “Hypostasized beneath neoliberalism, a global psychopharmaceutical industry *compels* targeted consumers to know about and from a space of impairment: ‘Ask your doctor,’ Big Pharma instructs the consumer, ‘if Cymbalta is right for you’” (128). Within a neoliberal health paradigm, “right for you” invariably entails treatment that allows a return to or intensification of work. McRuer and Johnson contend that working against these “[n]eoliberal disability epistemologies” involves “challenging subjects who confidently ‘know’ about ‘disability,’ as though it could be a thoroughly comprehended object of knowledge” (128, 130).

The attention deficit subject occupies a strange position in relation to neoliberal disability epistemologies because, as I discussed above, she is not yet fully known. Yet just as her attention deficit exists in relation to an idealized future productivity, so is her condition framed by anticipated scientific knowledge. While this knowledge is still pending, the attention deficit subject must prove her authenticity in order to access treatment. The blankness that frames the assembled fragments of *Why Did I Ever* may be seen to evoke the yet-to-be known that surrounds attention disorders. Rather than awaiting population by future medical certainties, however, Robison’s blank space is one element of her return to minimalist aesthetics—a return that deploys irony and parody to destabilize dichotomies between the inaccessible past of trauma and the yet-to-be-known future of ADD.

*Why Did I Ever’*s return to minimalism is self-conscious and interrogative, concerned above all to deflate the assumptions about knowledge embedded in the term’s diagnostic deployment. In a revelatory passage, Robison parodies Hemingway’s controlled economy with an oblique reference to the famous six-word story regularly, if inaccurately, attributed to him: “For sale: baby shoes, never worn.” Staying in a hotel for work, Money produces an alternative version of the story:

Left below the bed for me—a baby’s Stride Rite shoe.

I’m wondering about it, as I’m in bed and preparing for sleep, telling myself it’s fine that a terrific baby stayed here before me and there is no reason to believe the stay ended in tragedy just because the baby left behind its shoe. There. On to the next thing. I’m wondering, when do you ever see the truly attractive Christian men? I want to ride to church in a black van full of French-ski-champ-looking Christians. That, to me, would be the way to go. (114–15).

This is an ADD revision of Hemingway’s “tip of the iceberg” aesthetic principle, which, as Cynthia Whitney Hallett has observed, suggests that seven-eighths of the story lies beneath its surface.^12^ The 1980s account of minimalism’s attention deficit riffs off the iceberg metaphor: for the likes of Bell, minimalist writing is like a fraudulent iceberg that advertises its tip but yields nothing under the sea’s surface. Robison’s version of “baby shoes” refuses the suggestive restraint of the iceberg. It develops instead a digressive tenor that both intensifies and obscures the association of the baby shoe with death. If the “original” six-word story relies on the expectation of interpretive universality for its affective charge, Robison’s new version pierces its allusive certainty and queries what an abandoned baby shoe can really tell us about the fate of its wearer.

This deflationary rewriting, from one perspective, wholly embraces the kind of minimalism caricatured by Bell or Aldridge, in which the project of interpretation has been abandoned for lack of evidence. The “original” story yields an impression of certainty: we infer confidently that a baby has died. Robison’s parody, in contrast, emphasizes uncertainty: we can never know what happened to the baby, just as we can never reach a final, exhaustive explanation for Money’s behavior. Under the minimalism of descriptive psychiatry, Robison’s hyperactive psychological response to the baby shoe would be read as a meaningless symptom of her ADD rather than a meaningful symptom of her traumatized preoccupation with violence and death. Indeed, *Why Did I Ever* holds these two possibilities in suspension: Money has ADD *and* she is afraid of violence and death in the wake of her son’s ordeal.

I refer to the “original story” in quotation marks because even though it is popularly attributed to Hemingway, “For sale: baby shoes, never worn” has no definitive source. Robison’s repurposing of this anonymously authored story is part of the novel’s broader preoccupation with the role of copying and fakery in both aesthetic and clinical judgment. Critics of minimalism often shared an anxiety that the mode might be fraudulent: a hoodwink designed to trick the critic. This anxiety is at work in Broyard’s review of Robison, in which he wonders whether she might be fooling him—whether her work’s small scale might be a sign of aesthetic poverty rather than innovative constraint. *Why Did I Ever* wryly refers to such critical anxieties by making Money a keen art forger, as we learn in Episode 14: “For my living room I have forged three paintings and signed them all ‘Robert Motherwell.’ The paintings aren’t that successful really as I went too fast. They might fool a rich fellow who doesn’t expect to see a fake if anyone like that ever comes over here” (5).

This passage makes it clear that Money’s forgeries are not serious attempts to make money; later in the same episode, we learn that her faked Thomas Mann inscription in a copy of *Buddenbrooks* (1901) reads, improbably, “Party girl. Bring back my VCR” (5). Her copies appear as a form of research—an attempt to probe notions of authenticity as they operate in both aesthetic economies and medical ones. When Money’s daughter Mev confides that she has been presenting a soft drink instead of a urine sample at the methadone clinic, Money reassures her, “You won’t get caught” (2). Another chapter ends, “I’m admiring this letter I forged from the IRS. It reads: ‘You are paid in full’” (88). Holding institutionally sanctioned forms of evidence up for mockery, these passages underline Money’s refusal, or inability, to conform to the ideal model of the socially responsible and economically productive ADD patient.

Money’s friend Hollis critiques her Rothko: “What’s missing here is a focal point … Something for our eyes to fix on, finally, and rest upon. Something we end up gazing at” (6). If the charge recalls literary critical accusations of minimalist literature’s lack of focus, then Money’s response—“It’s! A! Copy!”—takes refuge in literary minimalism’s allegedly fatal weakness: its formulaic flatness. Money compares her Rothko forgery favorably to the real thing: “I’m fairly proud of the Rothko I forged for my bedroom. Whereas the blacks in the paintings at the Rothko Chapel can look a little steely and cold, my blacks are rich with the colors of hot embers and dark earth” (5). While this passage pokes fun at Money—does she really think she is better than Mark Rothko?—it also draws out a tension between notions of minimalism as a formulaic and cold aesthetic mode and *Why Did I Ever’*s subversion of these tropes. In fact, Money’s ADD manifests as a failure to make accurate copies. She admits that she “went too fast” with her Motherwell, and her version of the baby shoes story is not really a copy at all, partly because its author is unknown and partly because she so decisively transforms the story’s style. Dispelling the critical equation of attention disorders and formulaic art, Money cannot make the perfect copy if she tries.

The novel’s references to visual art might especially be read in light of Robison’s complaint that the term *minimalism* was misappropriated from painting. Despite Robison’s stated resistance to comparisons between literary minimalism and minimal art, the modular form of *Why Did I Ever* does recall visual works of the 1960s. The serial structure of Robison’s two post-2000 novels may very well bear a comparison to minimal art more readily than her canonical minimalist stories of the 1970s and 1980s. Like the serial installations of Donald Judd, Carl Andre, or Sol LeWitt, *Why Did I Ever* lacks a center: Robison has averred that “if you read the pages in reverse order, they work about the same” (M. Murray). With the modularity of *Why Did I Ever*, Robison self-consciously rises to the unstated challenge posed by the “minimalist” label that has chased her throughout her career: Is it possible, the novel implicitly asks, to produce a work of literature that can feasibly bear the same descriptor as works of minimal art? In the very act of mimicking it, however, Robison distances her work from minimal art’s nonmimetic commitments. With this paradoxical move, Robison addresses questions of transmedial commensurability: Money’s bad copies are, after all, only accessible through the written word.

These tensions are part of *Why Did I Ever’*s broader engagement with the problem of description. Rewriting Hemingway, forging Mann’s signature, and copying a Rothko are all ways of calling into question the critical caricature of flat minimalist verisimilitude: to merely redescribe the world, Robison implies, is never so easy. An exchange between Money and her boyfriend, Dix, brings this point home:

  “You’re saying?” Dix asks me.

  “What I’m saying,” I tell him.

  “But you mean?” he asks.

  “Same as the words mean,” I say. (54)

This dismissal of the very possibility of interpretation emerges again in the novel’s representation of Money’s therapeutic treatments for ADD, which take the notion of tautological description to the extreme. In a session with her therapist, she questions the purpose of the therapeutic writing tasks she has been assigned:

“No more journal,” I tell him. “I’m never bringing it to therapy again. All my time, any hour, any day of the week, is wasted. Pointless to record where or how.”

“Nor am I keeping any more organizational lists. I’ll show you why,” I say. “Here, you can read this. Its reminders are, one, ‘sweater’, two, ‘read newspaper.’

“I can’t,” I say, crumpling the page, “be this pitiful.” (37–38)

But Money does attempt some of these organizational strategies. She labels everything in the kitchen—“SINK, COUNTER, CABINETS, CLOCK, DOOR, REFRIGERATOR”—but still finds she can’t remember where to store a bag of potatoes and ends up putting them out in the yard (102).

In these episodes, organizational strategies for people with ADD evoke the harshest critical caricatures of Robison and her peers during the 1980s. Money’s ADD management strategies, and her curt reference to what “the words mean,” result in a form of writing that leaves no room for the work of interpretation (of course a sink is a sink). Yet if Money’s actions pastiche what Barth calls minimalism’s childlike “Dick-and-Jane” mode, they are also a serious negotiation with the no man’s land between the absurdity of descriptive mimesis and the impossibility of interpretation. In a doomed attempt to organize her domestic life, Money tries color-coding her house by painting every object, even her computer keyboard. She admits, “I shouldn’t have coated gold on the numeric keys. The alphabet I can touch-type but the ampersand is where in the hell?” (55)

In “Building a Better Description,” Love, Best, and Marcus urge a reassessment of descriptive tautology with the argument that “[i]f we free ourselves from the demand that everything be related to a grand theory or yield surplus knowledge, we might come to see even tautological description in a better light.” Such an embrace of tautology, they add, might remake description as a “noninstrumental accumulation of particulars with no immediately clear purpose” (14). Despite this appeal to noninstrumentality, the article moves on the same page to endorse description for its attention-enhancing qualities.^13^ Robison’s version of tautological description is far removed from this one: for Money, it is one thing to call a sink a sink but quite another to remember where the potatoes are stored. If tautological description improves attention for Love, Best, and Marcus, and marks out the expandable limits of psychiatric knowledge in the *DSM*, in *Why Did I Ever*, it emblematizes the failure of therapy and the persistence of attention deficit disorder.

At the same time, however, the novel attests to the impossibility of pure mimetic description—and, in doing so, it makes ADD the site on which minimalist sparseness and hyperactive excess converge. The novel’s representation of Money’s writing life points to the limitations of the concept of *deficit* for an understanding of ADD writing—and, perhaps, for an understanding of the condition itself. Trying to hold onto a job on a terrible big-budget film called *Bigfoot*, Money is instructed by her antagonist, producer Belinda, not to “get creative” (45). She makes a resolution: “I won’t cut anything, I’ll just add” (85). Committing to this decision by adding an array of absurd details to the script, Money eventually takes her additive writing philosophy to the limits of sense: “It isn’t anything but as I’m writing my notes for tomorrow I fill up a page and don’t turn to a new page. I just press down hard with my pen and write over the top of what I’ve already written” (78). Money’s refusal to cut represents the antithesis of Lish’s editing practice. These descriptions of Money’s writing align *Why Did I Ever* with *Tell Me*: both works undo—whether symbolically or literally—Lish’s commitment to spare writing and represent Robison’s project as a reversal of canonical minimalist style. They also suggest that any attempt to define “ADD aesthetics” must contend with the condition’s resistance to diagnostic certainty.

In drawing attention to the limits of knowledge about attention disorders, Robison challenges some of the presumptions of the descriptive psychiatry that shaped post-1980 editions of the *DSM*. The *DSM*’s concerted marking of its own epistemological limits can be said to deploy a form of minimalism, but the manual’s resistance to interpretation constitutes a paradox. By flagging up the absence of secure, empirical evidence, the handbook leaves a blank space for a speculation relentlessly conditioned by a neoliberal imaginary of scientific certainty, enhancement, and productivity. *Why Did I Ever* destabilizes such neurobiological speculation through an insistent focus on Money’s subjective experience, which deprivileges institutional investments in static classification. The *DSM*’s minimalism hints at the limitlessness of the psychiatric knowledge that lies below the tip of the iceberg. In contrast, Robison writes in a spirit of cripistemological unknowing, holding the dual possibilities of trauma and ADD in suspension without making ADD’s mute symptoms the site of future empirical proof and correction.

Robison’s cripistemological writing has implications for practices of literary labeling too. The critics referred to in this article simultaneously deploy and disavow the term *minimalism*, because it—like ADD—seems to resist interpretive mastery. This kind of literary diagnosis secures the boundaries of the canon by stigmatizing forms of writing deemed to be in deficit. My reading of *Why Did I Ever* demonstrates that this mode of diagnostic reading is incompatible with the fact that attention disorders, like many other neurobehavioral conditions, can be lived without being fully known. Alert to this context, Robison’s cripistemological writing captures the everyday textures through which attention disorders are made, within and beyond psychiatric and literary description. 

## Footnotes

1 This article has benefited from the generous feedback of three anonymous peer reviewers and audiences at King’s College London, the University of East Anglia, and Birkbeck, University of London. Thanks also to James Mansfield for reading and commenting on multiple drafts. This work was funded by an ISSF award from Birkbeck and the Wellcome Trust (Grant Number 204770/Z/16/Z).

2 See Peter Conrad and Deborah Potter, “From Hyperactive Children to ADHD Adults: Observations on the Expansion of Medical Categories,” *Social Problems*, vol. 47, no. 4, 2000, pp. 559–82.

3 The notion that patterns of attention shape, and are shaped by, practices of reading and writing is by no means confined to the late twentieth century, of course. Nicholas Dames, for instance, has argued that the physiological novel theory of the nineteenth century was particularly marked by a curiosity about the interactions of literature and attention. In *The Physiology of the Novel* (2007), Dames notes that a “good way of categorizing a critical school or critical practice is to define the pitch, intensity, and duration of readerly attention that it employs and seeks to make normative” (13). The twentieth-century critics I examine here were interested in similar questions, but their focus was trained specifically on the waning attention span: they were concerned that minimalist literature made normative a pathologically deficient form of attention.

4 For an account of Lish's compositional “logic” and its influence on American fiction beyond the minimalist paradigm, see David Winters, “Theory and the Creative Writing Classroom: Conceptual Revision in the School of Gordon Lish,” *Contemporary Literature*, vol. 57, no. 1, 2016, pp. 111–34. Winters notes, “If Lish's reception as a minimalist reflects a misleadingly small sample size, it also impedes a complete understanding of his teaching” (115).

5 A prominent instance of this reading is Mark McGurl’s comment on the way women minimalists “reverse the seeming affinity of minimalism with silent masculinity, making it work to record and manage the trauma of female experience” (*The Program Era: Postwar Fiction and the Rise of Creative Writing* [2009], 69). Andrew Hoberek also highlights trauma in his discussion of minimalism’s affinities with the Vietnam novel, see “Foreign Objects, or, DeLillo Minimalist,” *Studies in American Fiction*, vol. 37, no. 1, 2010, pp. 101–25. Doherty offers a different interpretation of the relationship between minimalism and Vietnam, positioning “state-supported minimalism as a means of restoring collective identity, not simply as an expression of personal psychological trauma” (90).

6 Hoberek’s exemplary summary (pp. 101–25) states that “minimalism arose to challenge the prominence of the big, nonrealistic postmodern novel associated with writers like Thomas Pynchon, John Barth, and Robert Coover” (103).

7 Here Puar quotes Jack Halberstam’s contention that “[a]ny cripistemology worth its name should identify modes of not knowing, unknowing, and failing to know” (qtd. in Johnson and McRuer, “Proliferating Cripistemologies” 152).

8 Attention and distraction are key concerns in maximalist postmodern literature too. Though I lack the space to explore them here, maximalist texts, including Franzen’s *The Corrections* (2001) and Wallace’s *Infinite Jest* (1996) and unfinished novel *The Pale King* (2011), all focus on forms of cognitive enhancement and deficit correction. This indicates the way the minimalism/maximalism binary in contemporary US literature structures concerns about the malleability, whether chemical or cultural, of attention and distraction.

9 This move anticipates Carver’s *Beginners* (2009), a posthumously published volume consisting of the unedited manuscript of his story collection *What We Talk About When We Talk About Love* (1981), which was heavily edited by Lish. However, the stories published in *Tell Me* are not unedited—rather, they replace the Lish-edited versions with the Angell-edited versions—and so the book does not evoke notions of authorly authenticity in the manner of *Beginners*.

10 See M. Murray, “Interview with Mary Robison”; Barth, “A Few Words About Minimalism.”

11 Barth taught and mentored Robison and has praised her work in unequivocal terms from her career’s beginning.

12 Hallett, in *Minimalism and the Short Story* (1999), draws a contrast between Hemingway's iceberg principle and “the Minimalist artist’s ‘whole ice cube’ effect, with which all that is seen is all that there is” (1). The modular structure of much minimal artwork complicates Hallett's distinction between the ice cube and the iceberg: the repeated forms of a Judd installation, for instance, contain the ever-present possibility of further replication, suggesting that the ice cube might not be a self-contained whole after all. I wish to thank the anonymous reviewer who raised this point about minimal art and process.

13 Since the 2001 publication of *Why Did I Ever*, a new public narrative of crisis around attention and distraction has gained prominence in the context of the rise of digital media. These narratives are, undoubtedly, an important context for Marcus, Love, and Best’s appeals to description as a strategy for honing readerly attention. See also Felski’s endorsement of phenomenology’s “willingness to attend rather than to analyze” (107).
